# Supplementary material for: Increased Prevalence of Myocardial Infarction and Stable Stroke Proportions in Patients with Inflammatory Bowel Diseases in Quebec in 1996–2015
Source: J Clin Med. 2022 Jan 28;11(3):686. doi: 10.3390/jcm11030686 (PMC8837182; doi:10.3390/jcm11030686)
Supplement: Supplementary file 1 [file jcm-11-00686-s001.zip › jcm-1506299-supplementary.pdf]

Supplementary Tables

Table S1. Association between risk factors and Crohn's diseases and myocardial infarction prevalence in total inflammatory bowel diseases population.

CD/MI Prevalence total population [n]	Univariate Analysis			Multivariate Analysis		
**Variables**	**OR**	**95% CI**	**p-value**	**OR**	**95% CI**	***p*-value**
Age	1.077	(1.072-1.083)	<0.0001	1.046	(1.04-1.052)	<0.0001
Gender	2.022	(1.724-2.371)	<0.0001	1.945	(1.65-2.292)	<0.0001
Diabetes	2.808	(2.321-3.397)	<0.0001	1.586	(1.286-1.957)	<0.0001
Hypertension	3.776	(3.114-4.58)	<0.0001	2.549	(2.06-3.155)	<0.0001
Hyperlipidaemia	3.694	(3.098-4.404)	<0.0001	2.838	(2.334-3.452)	<0.0001

Note: Univariate analysis adjusted for age and sex. CD: Crohn's disease, MI: myocardial infarction, OR: odds ratio, CI: confidence interval; *n* = 35,985.

Table S2. Association between risk factors and Crohn's diseases and stroke prevalence in total inflammatory bowel diseases population.

CD/STROKE Prevalence total population [n]	Univariate Analysis			Multivariate Analysis		
**Variables**	**OR**	**95% CI**	**p-value**	**OR**	**95% CI**	***p*-value**
Age	1.082	(1.076-1.089)	<0.0001	1.051	(1.051-1.065)	<0.0001
Gender	1.107	(0.92-1.331)	0.2789	0.886	(0.886-1.289)	0.4892
Diabetes	1.817	(1.447-2.281)	<0.0001	0.952	(0.952-1.577)	0.1151
Hypertension	2.768	(2.23-3.43)	<0.0001	1.955	(1.955-3.167)	<0.0001
Hyperlipidaemia	2.103	(1.731-2.557)	<0.0001	1.549	(1.55-2.41)	<0.0001

Note: Univariate analysis adjusted for age and sex. CD: Crohn's disease, OR: odds ratio, CI: confidence interval; *n* = 35,985.

Table S3. Association between risk factors and Crohn's diseases and myocardial infarction incidence in total inflammatory bowel diseases population.

CD/MI Incidence total population [n]	Univariate Analysis			Multivariate Analysis		
**Variables**	**IRR**	**95% CI**	***p*-value**	**IRR**	**95% CI**	**p-value**
Age	1.0704	(1.063-1.077)	<0.0001	1.056	(1.04-1.064)	<0.0001
Gender	1.8482	(1.48-2.309)	<0.0001	1.7917	(1.433-2.239)	<0.0001
Diabetes	2.5306	(1.907-3.357)	<0.0001	1.883	(1.394-2.543)	<0.0001
Hypertension	2.2732	(1.738-2.972)	<0.0001	1.750	(1.312-2.335)	0.0001
Hyperlipidaemia	1.9692	(1.527-2.538)	<0.0001	1.462	(1.112-1.922)	0.0064

Note: Univariate analysis adjusted for age and sex. CD: Crohn's disease, MI: myocardial infarction, IRR: incidence rate ratio, CI: confidence interval; *n* = 35,985.

Table S4. Association between risk factors and Crohn's diseases and stroke incidence in total inflammatory bowel diseases population.

CD/STROKE Incidence total population n	Univariate Analysis			Multivariate Analysis		
**Variables**	**IRR**	**95% CI**	***p*-value**	**IRR**	**95% CI**	***p*-value**
Age	1.0678	(1.058-1.077)	<0.0001	1.0678	(1.058-1.077)	<0.0001
Gender	1.083	(0.805-1.456)	0.5975	1.083	(0.805-1.456)	0.5975
Diabetes	1.485	(0.957-2.303)	0.077	--	--	--
Hypertension	1.368	(0.96-1.94)	0.08	--	--	--
Hyperlipidaemia	1.371	(0.97-1.933)	0.071	--	--	--

Note: Univariate analysis adjusted for age and sex. CD: Crohn's disease, IRR: incidence rate ratio, CI: confidence interval; *n* = 35,985.

Table S5. Association between risk factors and ulcerative colitis and myocardial infarction prevalence in total inflammatory bowel diseases population

UC/MI Prevalence total population n	Univariate Analysis			Multivariate Analysis		
**Variables**	**OR**	**95% CI**	***p*-value**	**OR**	**95% CI**	***p*-value**
Age	1.076	(1.07-1.081)	<0.0001	1.046	(1.04-1.053)	<0.0001
Gender	2.183	(1.855-2.569)	<0.0001	2.152	(1.82-2.545)	<0.0001
Diabetes	1.911	(1.579-2.313)	<0.0001	1.173	(0.952-1.446)	0.1345
Hypertension	3.463	(2.876-4.17)	<0.0001	2.297	(1.865-2.83)	<0.0001
Hyperlipidaemia	3.391	(2.86-4.02)	<0.0001	2.775	(2.294-3.358)	<0.0001

Note: Univariate analysis adjusted for age and sex. UC: ulcerative colitis, MI: myocardial infarction, OR: odds ratio, CI: confidence interval; *n* = 35,985.

Table S6. Association between risk factors and ulcerative colitis and stroke prevalence in total inflammatory bowel diseases population.

UC/STROKE Prevalence total population n	Univariate Analysis			Multivariate Analysis		
**Variables**	**OR**	**95% CI**	**p-value**	**OR**	**95% CI**	**p-value**
Age	1.091	(1.084-1.098)	<0.0001	1.073	(1.065-1.081)	<0.0001
Gender	1.405	(1.159-1.704)	0.0005	1.397	(1.149-1.699)	0.0008
Diabetes	1.821	(1.457-2.274)	<0.0001	1.376	(1.08-1.753)	0.0097
Hypertension	2.334	(1.872-2.909)	<0.0001	1.882	(1.469-2.412)	<0.0001
Hyperlipidaemia	1.741	(1.43-2.12)	<0.0001	1.516	(1.215-1.892)	0.0002

Note: Univariate analysis adjusted for age and sex. UC: ulcerative colitis, OR: odds ratio, CI: confidence interval; *n* = 35,985.

Table S7. Association between risk factors and ulcerative colitis and myocardial infarction incidence in total inflammatory bowel diseases population.

UC/MI Incidence total population *n*	Univariate Analysis			Multivariate Analysis		
**Variables**	**IRR**	**95% CI**	**p-value**	**IRR**	**95% CI**	**p-value**
Age	1.0716	(1.064-1.08)	<0.0001	1.0591	(1.05-1.068)	<0.0001
Gender	1.9076	(1.52-2.391)	<0.0001	1.8903	(1.505-2.373)	<0.0001
Diabetes	1.4991	(1.113-2.018)	0.0077	1.2456	(0.916-1.693)	0.1614
Hypertension	1.7836	(1.378-2.308)	<0.0001	1.5605	(1.184-2.056)	0.0016
Hyperlipidaemia	1.5728	(1.23-2.009)	0.0003	1.3291	(1.024-1.725)	0.0324

Note: Univariate analysis adjusted for age and sex. UC: ulcerative colitis, MI: myocardial infarction, IRR: incidence rate ratio, CI: confidence interval; *n* = 35,985.

Table S8. Association between risk factors and ulcerative colitis and stroke incidence in total inflammatory bowel diseases population.

UC/STROKE Incidence total population *n*	Univariate Analysis			Multivariate Analysis		
**Variables**	**IRR**	**95% CI**	**p-value**	**IRR**	**95% CI**	**p-value**
Age	1.0885	(1.077-1.099)	<0.0001	1.0802	(1.067-1.093)	<0.0001
Gender	1.2761	(0.951-1.711)	0.1036	1.2584	(0.936-1.71)	0.1277
Diabetes	1.7325	(1.198-2.504)	0.0035	1.594	(1.091-2.327)	0.0157
Hypertension	1.5407	(1.099-2.158)	0.012	1.4107	(0.999-1.992)	0.0506
Hyperlipidaemia	1.0756	(0.78-1.483)	0.6567	--	--	--

Note: Univariate analysis adjusted for age and sex. UC: ulcerative colitis, IRR: incidence rate ratio, CI: confidence interval; *n* = 35,985.

Table S9. STROBE Statement—Checklist of items that should be included in reports of ***cohort studies.***

	Item No	Recommendation	Page number
**Title and abstract**	1	(*a*) Indicate the study's design with a commonly used term in the title or the abstract	1
		(*b*) Provide in the abstract an informative and balanced summary of what was done and what was found	1
**Introduction**			
Background/rationale	2	Explain the scientific background and rationale for the investigation being reported	1-2
Objectives	3	State specific objectives, including any prespecified hypotheses	2
**Methods**			
Study design	4	Present key elements of study design early in the paper	2
Setting	5	Describe the setting, locations, and relevant dates, including periods of recruitment, exposure, follow-up, and data collection	2-4
Participants	6	(*a*) Give the eligibility criteria, and the sources and methods of selection of participants. Describe methods of follow-up	2-4
		(*b*) For matched studies, give matching criteria and number of exposed and unexposed	NA
Variables	7	Clearly define all outcomes, exposures, predictors, potential confounders, and effect modifiers. Give diagnostic criteria, if applicable	2-4
Data sources/ measurement	8*	For each variable of interest, give sources of data and details of methods of assessment (measurement). Describe comparability of assessment methods if there is more than one group	2-4
Bias	9	Describe any efforts to address potential sources of bias	8
Study size	10	Explain how the study size was arrived at	2
Quantitative variables	11	Explain how quantitative variables were handled in the analyses. If applicable, describe which groupings were chosen and why	3-4
Statistical methods	12	(*a*) Describe all statistical methods, including those used to control for confounding	3-4
		(*b*) Describe any methods used to examine subgroups and interactions	3-4
		(*c*) Explain how missing data were addressed	3-4
		(*d*) If applicable, explain how loss to follow-up was addressed	NA
		(*e*) Describe any sensitivity analyses	NA
**Results**			
Participants	13*	(a) Report numbers of individuals at each stage of study—eg numbers potentially eligible, examined for eligibility, confirmed eligible, included in the study, completing follow-up, and analysed	4
		(b) Give reasons for non-participation at each stage	NA
		(c) Consider use of a flow diagram	NA
Descriptive data	14*	(a) Give characteristics of study participants (eg demographic, clinical, social) and information on exposures and potential confounders	4-5
		(b) Indicate number of participants with missing data for each variable of interest	NA

		(c) Summarise follow-up time (eg, average and total amount)	follow -up time max 20 years, median 10 years
Outcome data	15*	Report numbers of outcome events or summary measures over time	5-7
Main results	16	(*a*) Give unadjusted estimates and, if applicable, confounder-adjusted estimates and their precision (eg, 95% confidence interval). Make clear which confounders were adjusted for and why they were included	5-7
		(*b*) Report category boundaries when continuous variables were categorized	5-7
		(*c*) If relevant, consider translating estimates of relative risk into absolute risk for a meaningful time period	5-7
Other analyses	17	Report other analyses done—eg analyses of subgroups and interactions, and sensitivity analyses	5-7
**Discussion**			
Key results	18	Summarise key results with reference to study objectives	7-8
Limitations	19	Discuss limitations of the study, taking into account sources of potential bias or imprecision. Discuss both direction and magnitude of any potential bias	8
Interpretation	20	Give a cautious overall interpretation of results considering objectives, limitations, multiplicity of analyses, results from similar studies, and other relevant evidence	7-8
Generalisability	21	Discuss the generalisability (external validity) of the study results	8
**Other information**			
Funding	22	Give the source of funding and the role of the funders for the present study and, if applicable, for the original study on which the present article is based	10

*Give information separately for exposed and unexposed groups. Note: An Explanation and Elaboration article discusses each checklist item and gives methodological background and published examples of transparent reporting. The STROBE checklist is best used in conjunction with this article (freely available on the Web sites of PLoS Medicine at http://www.plosmedicine.org/, Annals of Internal Medicine at http://www.annals.org/, and Epidemiology at http://www.epidem.com/). Information on the STROBE Initiative is available at http://www.strobe-statement.org.
